# Lymphocyte reactivity in patients with carcinoma of the breast and large bowel.

**DOI:** 10.1038/bjc.1975.128

**Published:** 1975-07

**Authors:** J. J. Miller, P. R. Gaffney, J. A. Rees, M. O. Symes

## Abstract

The reactivity of lymphocytes from patients with either carcinoma of the breast or large bowel has been studied using the human to mouse normal lymphocyte transfer (NLT) reaction. It was found that, in the case of breast cancer, there was a direct correlation between the clinical stage and a reduced NLT reaction. Only patients with regional lymph node or generalized metastases showed significantly reduced lymphocyte reactivity. However, in the case of large bowel cancer there was a generalized reduction in NLT reactivity which was independent of the clinical stage. Incubation of lymphocytes from individuals without neoplastic disease in serum or plasma from breast cancer patients, showing reduced NLT reactivity, resulted in a reduced NLT reaction. This appears to be indicative of the presence of circulating "blocking factor" in such patients.


*Br. J. Cancer* (1975) **32**, 16

### LYMPHOCYTE REACTIVITY IN PATIENTS WITH CARCINOMA OF THE BREAST AND LARGE BOWEL

J. J. MILLER, P. R. GAFFNEY*, J. A. REES AND M. O. SYMES

*From the Department of Surgery, University of Bristol, The Medical School, University Walk, Bristol BS8 1TD*

Received 3 February 1975. Accepted 9 April 1975

**Summary.—The reactivity of lymphocytes from patients with either carcinoma of the breast or large bowel has been studied using the human to mouse normal lymphocyte transfer (NLT) reaction. It was found that, in the case of breast cancer, there was a direct correlation between the clinical stage and a reduced NLT reaction. Only patients with regional lymph node or generalized metastases showed significantly reduced lymphocyte reactivity. However, in the case of large bowel cancer there was a generalized reduction in NLT reactivity which was independent of the clinical stage. Incubation of lymphocytes from individuals without neoplastic disease in serum or plasma from breast cancer patients, showing reduced NLT reactivity, resulted in a reduced NLT reaction. This appears to be indicative of the presence of circulating " blocking factor " in such patients.**

HUGHES and Mackay (1965) demonstrated a decrease in skin reactivity to tuberculin, in patients with carcinoma of the breast, in whom the disease was no longer localized. Roberts (1974) skin tested breast cancer patients with the antigen Varidase which is a mixture of streptokinase and streptodornase. It was found that reactivity was reduced even in patients with localized disease. This finding was supported by investigations on lymphocyte reactivity *in vitro*. Whittaker and Clark (1971) and Watkins (1973) demonstrated diminished lymphocyte responsiveness to phytohaemagglutinin in early breast cancer patients. However, whilst the stage at which immunodepression was first manifest is disputed, all authors agree that it is progressive with advancing disease.

A similar situation obtains in carcinoma of the rectum where the skin reactivity of patients to the antigen 2,4-dinitrochlorobenzene is reduced in direct proportion to the clinical stage of the disease (Bone and Camplejohn, 1973). It thus seemed of interest to evaluate further the lymphocyte reactivity, *in vivo*, of patients with these types of malignant neoplasia. To this end the human to mouse normal lymphocyte transfer (NLT) reaction (Rees and Symes, 1973) has been employed.

### MATERIALS AND METHODS

### *Clinical material*

A total of 38 patients with carcinoma of the breast and 18 with carcinoma of the large bowel have been studied. All cases were staged according to the TNM classification (Bailey and Love, 1971; Wood, 1971). In addition, as control material, 15 patients with benign breast neoplasms and 17 post-surgical cases with non-neoplastic conditions were investigated. The above figures apply to patients from each of whom 10 million lymphocytes were transferred to a mouse. The NLT reactivity of further smaller groups of patients was studied at the 5 million cell level.

* Present address: Department of Surgery, Banting Institute, 100 College Street, Toronto, Ontario, Canada.

*Normal lymphocyte transfer reaction*

On Day 0 lymphocytes were injected intradermally into the shaved flank of an outbred Ash Porton mouse. One such injection of 5 or 10 million cells in 0·1 ml was made for each patient, except in cases where the number of cells obtained allowed simultaneous testing at the 5 and 10 million cell level. The diameter of the NLT reaction in mm was measured on Day 2 using a caliper with a vernier scale. Two readings (to the nearest 0·5 mm) were taken at right angles and the result expressed as a mean. All lesions were measured by two independent observers. The lesion was sharply demarcated against the shaved skin of the mouse in the majority of cases. However, where the lesion was ill-defined, the area of the skin bearing the reaction was excised. The deep aspect of the reaction, which was invariably clearly seen, was then measured. To determine the reproducibility of the reaction for lymphocytes from a single individual 19 tests at a $10 \times 10^6$ lymphocyte level were made for a single healthy male. This gave a mean reaction size of $3{\cdot}15 \pm 0{\cdot}45$ (s.d.) mm which was suitably consistent.

*Separation of lymphocytes from venous blood*

In patients undergoing excision biopsy of a breast lump or mastectomy, 20 ml heparinized venous blood were obtained prior to the commencement of surgery. A similar sample of blood was also obtained from each patient with carcinoma of the large bowel, prior to surgery. Lymphocytes were separated by density gradient centrifugation after the blood had been layered on to a Ficoll–Isopaque column (Rees and Symes, 1973). Alternatively the blood was layered onto a Ficoll (9%, 24 parts) Triosil (34%, 10 parts) column. Equal volumes of Ficoll–Triosil and blood were then centrifuged at 2000 *g* for 20 min after which the lymphocytes were separated and washed as previously described (Rees and Symes, 1973).

*Studies on the effect of incubation, with plasma or serum from patients with metastatic breast cancer, on the reactivity of lymphocytes from healthy individuals*

In order to determine whether a lymphocyte blocking factor was present in patients with advanced breast cancer, plasma and serum from these individuals was incubated with lymphocytes from individuals free of neoplastic disease. The lymphocytes were obtained from the peripheral blood of a single healthy individual or from the spleens of patients undergoing splenectomy for benign oesophageal stricture.

In the case of a spleen, a nucleated cell suspension, in medium 199, was prepared by gently pressing and washing spleen slices through stainless steel sieves of decreasing porosity, 40, 100 and finally 200 mesh. The concentration of the suspension was adjusted to approximately 30 million cells per ml, and this was layered onto a Ficoll–Isopaque or Ficoll–Triosil mixture, as for venous blood.

In each experiment aliquots from the same lymphocyte suspension were incubated in some of the following: medium 199, plasma or serum from individuals without neoplasia, and plasma or serum from patients with metastatic breast cancer. All plasma or serum samples were heated to 56°C for half an hour prior to use. Each plasma or serum sample was diluted to one-fifth in 199 and incubated for 45 min at 37°C. Following incubation the lymphocytes were washed once in 199. Using each aliquot of lymphocytes, multiple NLT reactions were then performed.

*Statistical treatment of the data*

In a number of cases the reaction following lymphocyte transfer was recorded as " Nil ". Hence, in order to calculate the degree of significance between the NLT reactivity of cancer patients and controls, the non-parametric Rank–Sum Test (two populations), Hayslett (1968), was employed. In addition, the means and standard deviations of the reactions which could be measured were recorded.

The data on reactivity of lymphocytes following incubation in plasma did not contain any " Nil " readings, so the usual parametric tests for significance were employed. However, " Nil " readings were present following incubation in serum, so again the Rank–Sum Test was used.

### RESULTS

The lymphocyte reactivities of the several categories of individuals with

TABLE I.—*The Immunocompetence, as Assessed by the Human to Mouse Normal Lymphocyte Transfer Reaction, in Patients with Breast and Colon Carcinoma. In Each Case* 10 *Million Lymphocytes were injected I.D. and the Reaction Read on Day Two*

| | NLT reaction | | Rank–Sum test | | |
|---|---|---|---|---|---|
| | | | | Versus | |
| Category | No* | Mean diam. (mm) ± s.d. | No* | A | B |
| A. Surgical patients with non-malignant conditions | 17 | 2·85 ± 1·09 | 19 | | |
| B. Patients with benign breast neoplasms | 15 | 2·73 ± 0·93 | 15 | | |
| Patients with Ca breast | | | | | |
| C. $T_1No$ | 11 | 2·13 ± 0·94 | 11 | Z − 1·07NS | Z − 1·37NS |
| D. $T_{2-3}No$ | 10 | 2·71 ± 0·93 | 11 | NS | NS |
| E. $T_{2-3}N_2$ | 7 | 1·91 ± 1·09 | 10 | Z − 2·41<br>$P<0·01$<br>$<0·002$ | Z − 2·58<br>$P<0·01$<br>$<0·002$ |
| F. Multiple metastases | 2 | 1·15 | 6 | Z − 2·93<br>$P<0·01$<br>$>0·002$ | Z − 3·19<br>$P<0·002$<br>$>0·001$ |
| G. Ca colon or rectum | 12 | 2·42 ± 0·55 | 18 | Z − 2·07<br>$P<0·02$<br>$>0·01$ | |

* The difference between these two numbers equals the number of nil reactions.

and without neoplastic disease are presented in Table I. It may be seen that patients with breast cancer but without nodal metastases (categories C and D) showed no reduction in NLT reactivity when compared with either post-surgical cases with non-neoplastic conditions (category A), or patients with benign breast neoplasms (category B). This was true, irrespective of the size of the primary cancer, which was less than 2 cm diameter in category C and from 2–10 cm in category D. However, the occurrence of lymph node metastases (category E) led to a significant reduction in lymphocyte reactivity.

An even greater reduction in NLT reactivity was seen in breast cancer patients with multiple metastases (category F). In addition the NLT reactivity at the 5 million cell level, was compared between patients in categories B and F. The individual reaction sizes were: Category B, 1·3 : 2·5 : 2·5 : 2·8 : 2·8 mm. Category F, Nil : Nil : Nil : Nil : Nil : 0·5 mm. Using the Rank–Sum Test R = 45, B > F, $P < 0·04$.

Lymphocyte reactivity was reduced in a proportion of the patients with large bowel cancer. However, this reduction showed no correlation with TNM stage, Table II. If the NLT reactivity

TABLE II.—*A Synopsis of Patients with Carcinoma of the Colon or Rectum, in which Immunocompetence was Assessed by the Human to Mouse Normal Lymphocyte Transfer Reaction*

| | | | | NLT reaction (mm) | |
|---|---|---|---|---|---|
| No | Age | Sex | Stage | $5 \times 10^6$ | $10 \times 10^6$ |
| 1 | 77 | ♂ | $T_1NoMo$ | | Nil |
| 2 | 72 | ♂ | $T_3NoMo$ | | 2·0 |
| 3 | 78 | ♀ | $T_3NoMo$ | | 1·8 |
| 4 | 80 | ♂ | $T_3NoMo$ | | Nil |
| 5 | 74 | ♀ | $T_3NoMo$ | | 3·0 2·5 |
| 6 | 68 | ♀ | $T_3NoM_1$ | | Nil 2·8 |
| 7 | 64 | ♂ | $T_3NoM_2$ | | Nil |
| 8 | 64 | ♀ | $T_3N_1Mo$ | Nil | |
| 9 | 82 | ♀ | $T_3N_1Mo$ | | Nil |
| 10 | 60 | ♀ | $T_3N_1Mo$ | | 3·0 |
| 11 | 69 | ♀ | $T_3N_1Mo$ | | Nil |
| 12 | 64 | ♀ | $T_3N_1M_1$ | | 1·5 |
| 13 | 71 | ♀ | $T_3N\ M_1$ | | 2·8 |
| 14 | 84 | ♀ | $T_3N_1M_2$ | | 2·8 3·0 |
| 15 | 77 | ♂ | $T_3N_2M_1$ | | 1·8 |
| 16 | 65 | ♂ | $T_3N_2M_2$ | Nil | |
| 17 | 65 | ♂ | $T_3N_2M_2$ | | 2·0 |
| 18 | 70 | ♂ | $T_3N_2M_2$ | Nil | |
| 19 | 72 | ♂ | $T_4N_1Mo$ | Nil | |

of these cases as a whole, category G, is compared with that of patients in category A, Table I, then a significant reduction for the cancer patients is seen.

The effect of incubating lymphocytes from individuals without neoplasia in plasma of patients with metastatic carcinoma of the breast is shown in Table III. Plasma from two of three patients, B and C, significantly inhibited lymphocyte reactivity. The results of similar experiments involving serum rather than plasma are presented in Table IV. Again, sera from four different patients with metastatic breast carcinoma each significantly reduced lymphocyte reactivity in comparison with serum from individuals without neoplasia.

### DISCUSSION

In the present experiments, the reactivity of patients' lymphocytes is measured *in vivo* against a non-tumour-specific antigen mouse, which they had not previously encountered. Thus, a problem of interpretation which arises with *in vivo* immunocompetence testing using tuberculin or Varidase, is avoided.

The results reported in the case of breast cancer patients support the hypothesis that diminished lymphocyte reactivity follows the onset of malignant neoplasia. Thus, they are against the concept that carcinoma of the breast arises from a defect in immune surveillance.

The idea that depression of lymphocyte function is due to the presence of a tumour invites a consideration of mechanisms. The finding that NLT reactivity was reduced following incubation of lymphocytes from individuals without neoplasia in the presence of plasma or serum from cases of metastatic breast carcinoma suggests the involvement of a circulating factor. Similar diminution of lymphocyte responsiveness to PHA

TABLE III.—*The Effect on NLT Reactivity of Incubating† Peripheral Blood Lymphocytes or Splenic Lymphocytes with Plasma from Patients with Metastatic Breast Cancer*

| Type of lymphocyte | | Incubation: In medium 199 | Incubation: Healthy ♀ plasma | Patient's plasma: A | Patient's plasma: B | Patient's plasma: C |
|---|---|---|---|---|---|---|
| PBL | Ex. 1 | 2·5 (3)** ±0·16 | 2·6 (3)±0·16 | 2·8 (4)±0·13 | 2·1 (4)†† ±0·13 | |
| | Ex. 2 | | 3·9 (9)±0·24 | | | 2·8 (7)†† ±0·36 |
| Splenic | Ex. 1 | 2·7 (6)±0·23 | | 2·4 (6)±0·19 | | |
| | Ex. 2 | 2·4 (6)±0·23 | 2·0 (6)±0·23 | 1·5 (6)±0·23 | 2·2 (6)±0·23 | |
| | Ex. 3 | | 2·8 (3)±0·16 | 2·7 (3)±0·16 | 2·0 (3)†† ±0·16 | |

† Plasma dilution 1/5 45 min at 37°C.
** Mean diameter of NLT (mm) and ( ) number of observations ± S.E.
—— Significantly reduced $P < 0 \cdot 02$†† $P < 0 \cdot 05$†. Calculated by an analysis of variance on the total data for the experiments PBL Ex. 1 and splenic Ex. 2 and 3 by Student's $t$ test for PBL Ex. 2 and splenic Ex. 1.

TABLE IV.—*The Effect on NLT Reactivity of Incubating Peripheral Blood or Splenic Lymphocytes with Serum from Patients with Metastatic Breast Cancer*

| Type and number of lymphocytes | Incubation | NLT reactions (individual values) | Rank–Sum test *versus* incubation in normal serum |
|---|---|---|---|
| $7 \times 10^6$ PBL | Healthy serum | 2·8 : 3·0 : 3·0 : 3·3 : 3·8 | |
| | Patient 1 | 0·0 : 0·0 : 0·0 : 2·3 : 3·3 | R=18·5 $P<0 \cdot 05$ |
| | Patient 2 | 0·0 : 0·0 : 1·0 : 2·5 : 2·5 | R=15·0 $P<0 \cdot 03$ |
| $15 \times 10^6$ Splenic | Medium 199 | 2·3 : 2·3 : 2·3 : 2·8 : 2·8 | R=24 NS |
| | Healthy serum | 2·0 : 2·3 : 2·3 : 2·8 : 2·8 : 2·8 | |
| | Patient 3 | 0·0 : 0·0 : 0·0 : 0·0 : 0·5 : 0·5 | R=21 $P<0 \cdot 02$ |
| | Patient 4 | 0·0 : 0·0 : 0·0 : 0·0 : 0·5 : 1·0 | R=21 $P<0 \cdot 02$ |

in the presence of cancer patients' plasma was reported by Silk (1967). Gatti *et al.* (1970), Whittaker and Clark (1971) and Suciu-Foca *et al.* (1973), reported analogous findings following incubation of normal lymphocytes and sera from patients with solid tumours.

The nature of this circulating blocking factor is not known. However, Baldwin *et al.* (1973) incubated lymphocytes from rats immune to hepatoma, with a hepatoma-specific antigen extracted from this tumour. This resulted in abrogation of the cytotoxicity of the immune lymphocytes for the tumour. It is thus possible that the presence of circulating tumour antigen may explain the blocking of lymphocyte reactivity reported above. This hypothesis is being investigated by incubating normal lymphocytes with the high and low molecular weight fractions obtained following gel filtration on Sephadex G-150, of sera from breast cancer patients. The implication of tumour antigen would of course imply its ability to depress non-specific lymphocyte reactivity, as opposed responsiveness to tumour antigen. This has not, so far, been demonstrated, but the possibility seems worthy of investigation.

We thank Miss Benita Hall for her valuable technical assistance and Miss Heather Turner for preparing the typescript. We are much indebted to the Surgeons of the Avon Area Health Authority (Teaching) and the Royal Devon and Exeter Hospital for allowing us to study their patients.

This work was supported by a generous grant from the Cancer Research Campaign.

### REFERENCES

BAILEY, H. & LOVE, MC. N. (1971) *Short Practice of Surgery*, 15th Ed., revised A. J. H. RAINS, & W. M. CAPPER, London: H. K. Lewis. p. 44.

BALDWIN, R. W., PRICE, M. R. & ROBBINS, R. A. (1973) Significance of Serum Factors Modifying Cellular Immune Responses to Growing Tumours. *Br. J. Cancer* Suppl. I, **28**, 37.

BONE, G. & CAMPLEJOHN, R. (1973) The Role of Cellular Immunity in Control of Neoplasia. *Br. J. Surg.*, **60**, 824.

GATTI, R. A., GARRIOCH, D. B. & GOOD, R. A. (1970) Depressed PHA Responses in Patients with Non-lymphoid Malignancies. *Proc. Fifth Leucocyte Culture Conf.*, Ottawa. p. 339.

HAYSLETT, H. T. (1968) *Statistics Made Simple.* London: W. M. Allen. p. 189.

HUGHES, L. E. & MACKAY, W. D. (1965) Suppression of the Tuberculin Response in Malignant Disease. *Br. med. J.*, ii, 1346.

REES, J. A. & SYMES, M. O. (1973) An *in vivo* Test for the Immunocompetence of Human Lymphocytes. *Transplantation*, **16**, 565.

ROBERTS, M. M. (1974) Cellular Immunity in Breast Cancer. M.D. Thesis, University of Wales.

SILK, M. (1967) Effect of Plasma from Patients with Carcinoma on *in vitro* Lymphocyte Transformation. *Cancer, N.Y.*, **20**, 2088.

SUCIU-FOCA, N., BUDA, J., MCMANUS, J., THIEN, J. & REEMTSMA, K. (1973) Impaired Responsiveness of Lymphocytes and Serum Inhibitory Factors in Patients with Cancer. *Cancer Res.*, **33**, 2473.

WATKINS, S. M. (1973) The Effects of Surgery on Lymphocyte Transformation in Patients with Cancer. *Clin. & exp. Immunol.*, **14**, 69.

WHITTAKER, M. G. & CLARK, C. G. (1971) Depressed Lymphocyte Function in Carcinoma of the Breast. *Br. J. Surg.*, **58**, 717.

WOOD, D. A. (1971) Clinical Staging and End Results Classification: TNM System of Clinical Classification as Applicable to Carcinoma of the Colon and Rectum. *Cancer, N.Y.*, **28**, 109.


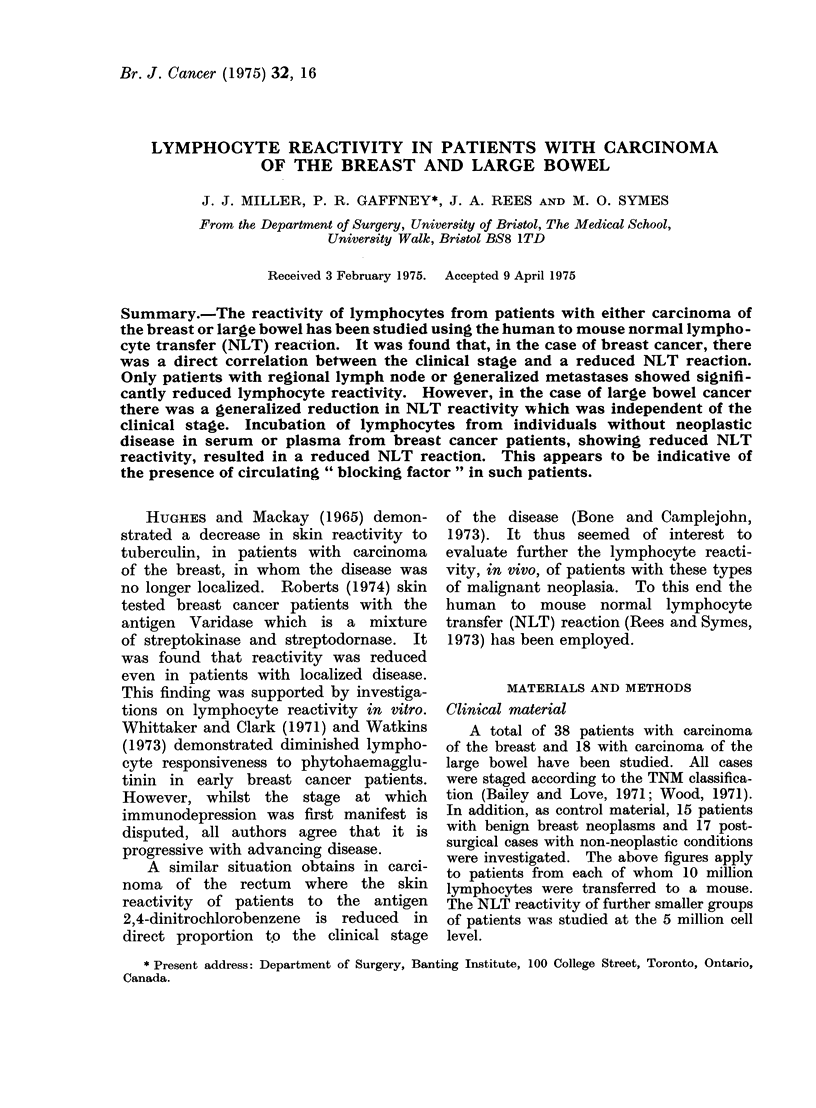

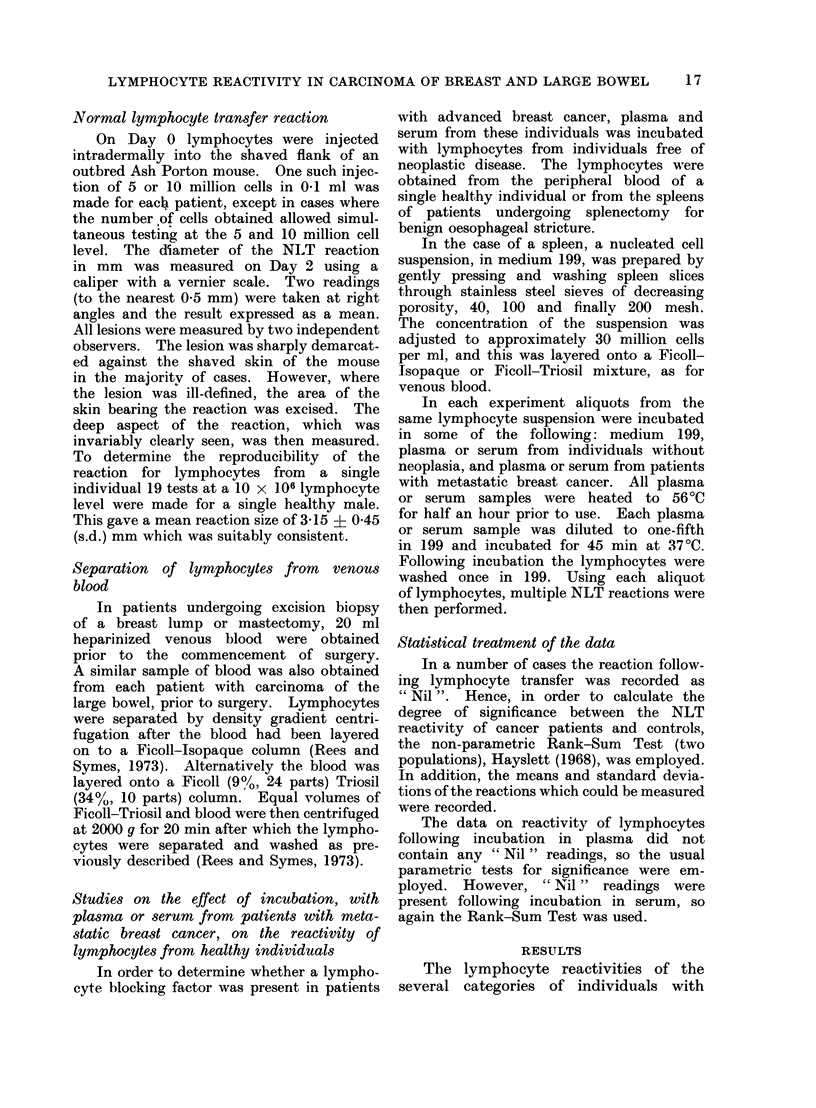

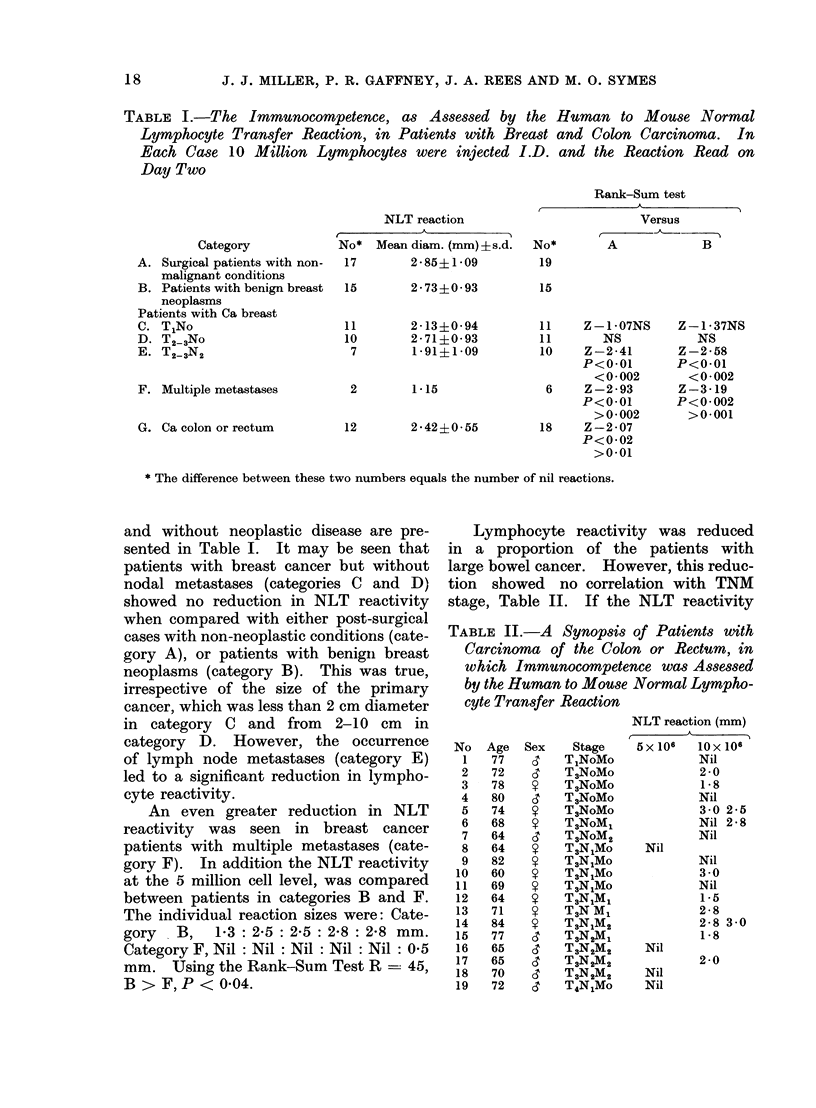

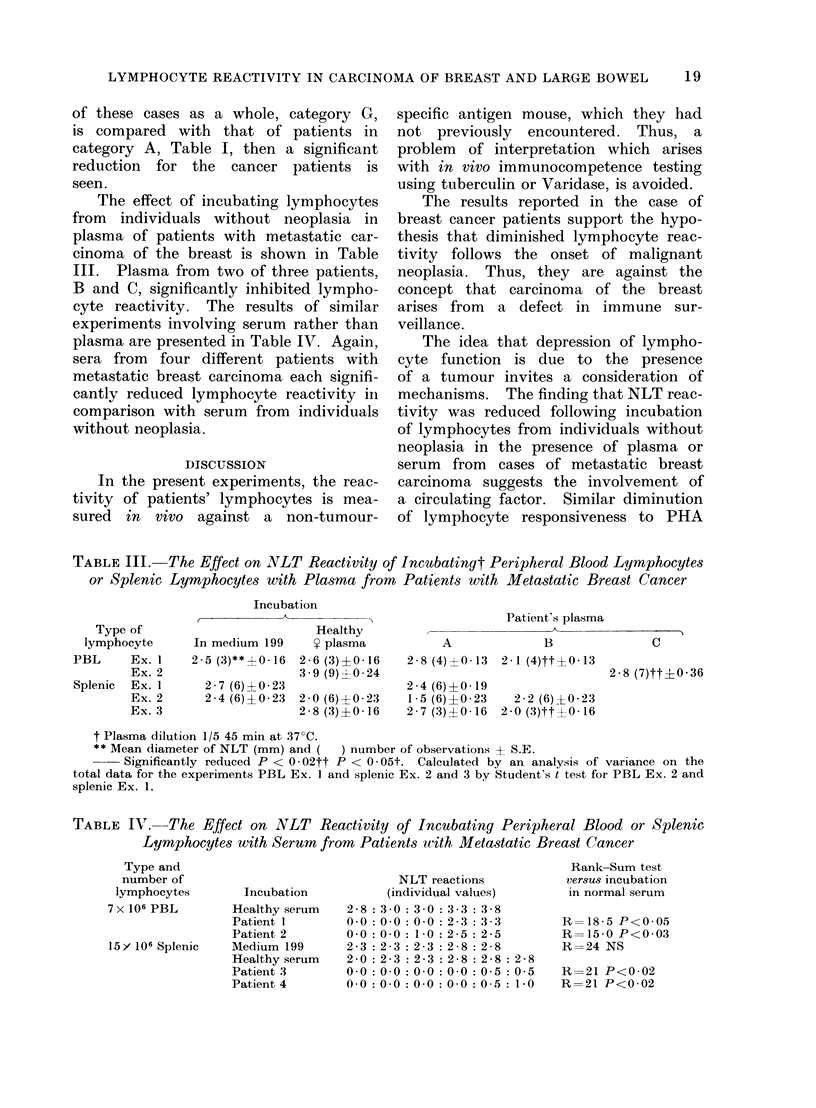

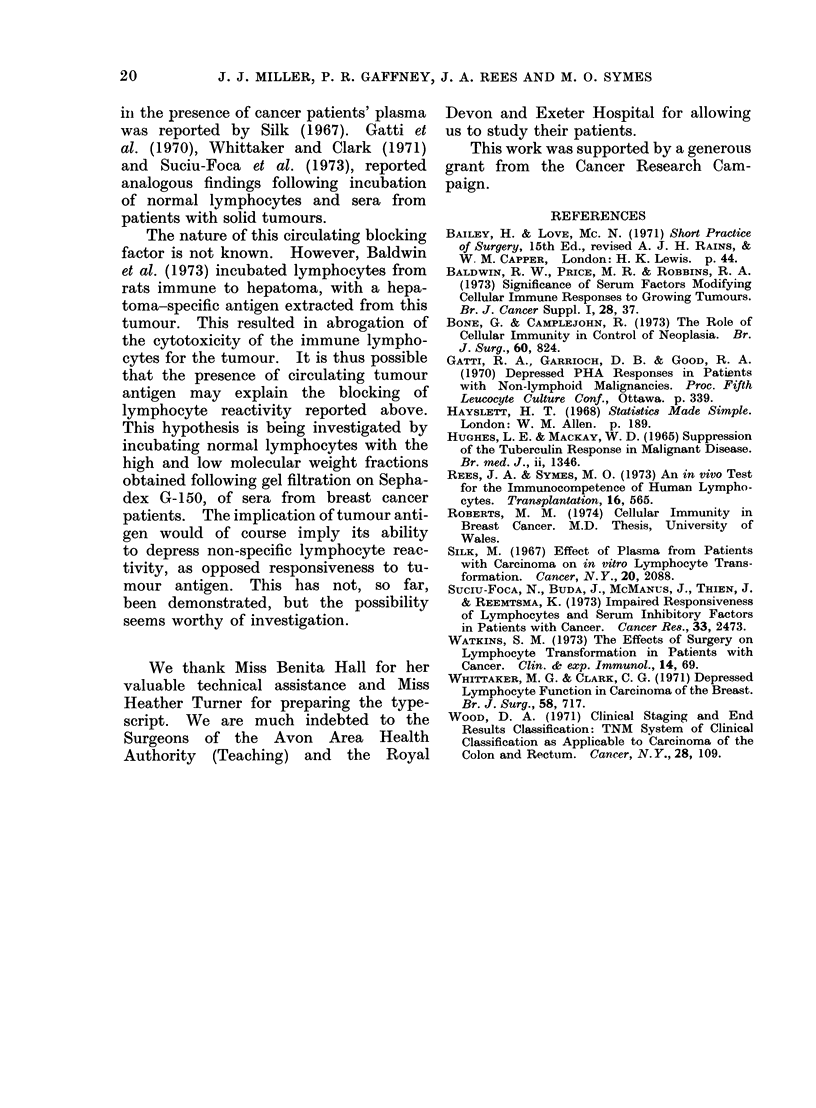


## References

[OCR_00673] Baldwin R. W., Price M. R., Robins R. A. (1973). Significance of serum factors modifying cellular immune responses to growing tumours.. Br J Cancer Suppl.

[OCR_00679] Bone G., Camplejohn R. (1973). The role of cellular immunity in control of neoplasia.. Br J Surg.

[OCR_00699] Rees J. A., Symes M. O. (1973). An in vivo test for the immunocompetence of human lymphocytes.. Transplantation.

[OCR_00709] Silk M. (1967). Effect of plasma from patients with carcinoma on in vitro lymphocyte transformation.. Cancer.

[OCR_00720] Watkins S. M. (1973). The effects of surgery on lymphocyte transformation in patients with cancer.. Clin Exp Immunol.

[OCR_00725] Whittaker M. G., Clark C. G. (1971). Depressed lymphocyte function in carcinoma of the breast.. Br J Surg.

[OCR_00730] Wood D. A. (1971). Clinical staging and end results classification: TNM system of clinical classification as applicable to carcinoma of the colon and rectum.. Cancer.

